# Extracts and Abstracts

**Published:** 1889-05

**Authors:** 


### EXTRACTS AND ABSTRACTS.

---

*The Treatment of Confirmed Catheter Life by a Permanent Perineal Opening.*—Walter Whitehead, F.R.C.S. Edin., Surgeon Royal Infirmary, Manchester, in a paper read before the Medical Society of London, March 14th, 1889, said:

I am quite aware that I am treading very closely upon the heels of those who have recently and very ably brought before the notice of this Society matter kindred in character to the subject of my communication, but I intend, so far as the interest of my subject will permit, to avoid going over any of the ground traversed on previous occasions.

My immediate object this evening is to submit for your consideration a plan which I have for some years found to be effectual, and I believe to be novel, in the treatment of men suffering from enlarged prostate who have not only arrived at catheter life, but have advanced to the stage when all prospects of their ever again being able to pass urine in the natural manner have to be abandoned as hopeless.

In these cases, Sir Henry Thompson, the veteran pioneer of all that relates to bladder surgery, advocates one of two plans: either the permanent retention of a tube through an opening established in the bladder above the pubes; or the plan of suspending the action of the bladder from seven to ten days by the temporary maintenance of a drainage-tube through the perineum. By the latter plan, he says, speaking of one case, "the relief was immediate and most remarkable," but he is compelled to admit that in every instance the patients have had sooner or later to resume their original practice of passing the catheter.

From Mr. Reginald Harrison's admirable Lettsomian lectures, I gather that he is in favor of drainage from the perineum. Previously we had those who were successful in many cases in relieving their patients by the constant use of retained catheters. As an alternative plan of treatment, we have also prostatotomy and prostatectomy, and more recently the suprapubic prostatectomy of Mr. McGill. Each of these plans appears to have fulfilled to a certain extent the purpose for which it was designed, but not one of them embraces the entire condition, or provides all that is desired in confirmed prostatic obstruction. For instance, the high outlet of a suprapubic opening leaves that particular part of the bladder which most requires drainage more remote than ever from the benefit of treatment. The perpetual presence of a perineal drain-tube is even less tolerable than a retained catheter, and a retained catheter can only be borne by a limited number of those who might otherwise be benefited by its use, and it is questionable whether the presence of these tubes does not as a rule aggravate the mischief and shorten life.

Mr. Harrison is singular, I believe, in the view he entertains that the prostate undergoes a diminution in size owing to the influence of the prolonged retention of a tube, and I am not aware that he claims to have had more than one case where the benefit from this plan has been more than partial and temporary.

Perineal prostatectomy appears to me an operation without any of the advantages of precision. You can neither regulate the amount, nor define the limits, of what you remove, and there is always the possibility that an undue mass may be taken away and permanent incontinence result.

McGill's suprapubic prostatectomy has, at least, the merit of affording the operator an opportunity of seeing the overgrowths, which project into the bladder, *in siiu*, but it affords no facilities for the removal of those growths which are confined to other portions of the prostate. The results of the cases reported by Mr. McGill were most excellent, but the number was small, and the conditions under which they were operated upon were exceptional. The mortality of suprapubic cystotomy in men over 50, it must be remembered, amounts to over 33 per cent., and this mortality is not likely, in the long run, to be lessened by the additional risk which must necessarily attend prostatectomy.

About six years ago I was more than ever impressed with the desirability of avoiding the pain and inconvenience which attend the constant passage of catheters; and I also became more familiar about this time with the facility with which a bladder could be entered for the purpose of digital exploration, and the idea occurred to me that at least three most important desiderata might be attained by a perineal urethrotomy. The bladder could be drained, it could be given necessary rest, and a considerable length of urethra would be relieved from the irritation of repeated catheterism. In consequence of the relapses which almost invariably followed the closure of the perineal wound and the reestablishment of the *status quo*, I further decided to maintain permanently the direct passage through the perineum. In the conception of this operation advantage was taken of the fact, which had been generally overlooked, that an opening in the membranous urethra does not involve, as many suppose, incontinence of urine.

I am aware that there are numerous instances on record where, after perineal cystotomy, the wounds have failed to heal, and a permanent fistula has remained; but in these cases the fistulæ have been accidental, and never, to my knowledge, have they been claimed as the result of a deliberate intention on the part of the operator. And, further, a perineal fistula in itself would afford no advantage; but if the fistula is made, and utilized in the manner I have to propose, then it becomes the key to the difficulty which attends the treatment of prostatic enlargement. I will now proceed, in the first place, to describe to you the operation itself, which differs in no essential detail from an ordinary median urethrotomy. A staff is passed into a previously moderately distended bladder. A lithotomy-knife, with the edge upwards, is plunged directly into the groove of the staff from a point an inch above the anus to a point a little in front of the prostatic apex. I prefer to enlarge the skin incision as I withdraw the knife, leaving the external wound, when finished, about an inch in length, and directed from the raphé toward the center of the space between the anus and the left tuber ischii. As the staff is withdrawn the finger is passed into the bladder when possible, and the opportunity is taken of making a digital exploration. When nothing is discovered admitting of immediate operation, the tube I have been at some pains in getting made to my satisfaction is introduced and maintained by a T-bandage, which I have found quite as effectual and much more comfortable to the patient than tapes. My preference for enlarging the external wound by one sweep of the knife during its withdrawal, in the place of cutting down on the staff by repeated incisions, is to secure a clean section and thus avoid an irregular wound, which gives greater facilities for the lodgment of discharges. The limited space between the point where the knife first enters the urethra and the rectum does not afford sufficient room for a wound which is required to remain permanent unless the incision be made obliquely in the manner described. The original opening in the skin should be made large enough at first, as it will then last for years without the necessity of dilating; but, if made too small, it may require enlargement within a few weeks of the operation. This, though a small matter to the surgeon, is generally a subject of considerable concern to the patient. It is the cicatricial contraction of the skin, and not of the deeper parts, which gives rise to this trouble.

Having obtained a short and wide passage of communication with the bladder, through which we experience no difficulty either in withdrawing the urine or in frequently injecting, with force and volume, large quantities of fluid, our next desire is generally to subdue the cystitis which usually exists, in as expeditious a manner as possible, and for this purpose there is nothing, as a rule, more simple or effectual than hot water. In order that this should be successful, the temperature should be gradually increased up to 120° F. Most patients can be induced by degrees to tolerate such a heat with comfort, and the higher the temperature, the greater the sedative effect.

There is nothing to be gained by using drugs simply for their germicide properties. What we require is something to scour from the tender mucous membrane every drop and particle of irritating matter. I have found creolin to excel every other drug in concealing, if not correcting, the putrid smell of some urines, and if the urine is loaded with tenacious viscid mucus, hot solutions of sea-salt appear to answer best.

One of the few drugs I have found of undoubted benefit in cystitis is the oxymuriate of bismuth. I was induced to try it from its well-known sedative influence upon the inflamed urethra. The plan of using it is to introduce a drachm, well agitated in four ounces of hot water, after the bladder has been thoroughly washed out. The ponderous character of the bismuth causes that which does not adhere to the mucous membrane to precipitate behind the prostate. Patients invariably express their satisfaction at the comfort they derive from this treatment. They state that the desire to micturate is not so frequent, and that the reflex pain along the urethra is diminished. It is necessary to remove the whole of the bismuth by irrigation before administering a second dose, as the bismuth undergoes chemical changes in the bladder, being most probably converted into a sulphide possessing no known therapeutic advantages.

I will now very briefly mention the particulars of three cases I have treated in this manner. I could supplement them with others were it desirable, some in which the results have been equally satisfactory, and others where the benefits have been limited to giving relief in hopeless cases during the last few weeks preceding death.

One of my earliest cases was a merchant, J. S., now 70 years old. I operated upon him in November, 1884, after he had been suffering for ten years from enlarged prostate and cystitis. He had all the usual symptoms in an aggravated form, and was compelled to abandon his business. The operation was perfectly successful, and he made a rapid convalescence. I lost sight of him for a number of years, owing to his removing out of town to reside in one of the suburbs. Within the last month I was successful in again finding him, and he reports himself as being quite cured, free from all discomfort, can retain his urine four hours, and attend to business and the Exchange regularly. He continues to withdraw his urine through his perineal aperture; and the urine is normal in every respect.

The next case I wish to mention is a very typical one, and will, I think, clearly illustrate all the features and advantages of the operation. Last June I was consulted by a gentleman, aged 63, who had suffered from symptoms of enlarged prostate for eight years. He was passing muco-purulent urine almost every quarter of an hour, night and day, and his general health was rapidly giving way under the constant suffering and loss of sleep. Being averse to any operation, he struggled against fate for five months, hoping against hope, and washing out his bladder with every possible solution until the climax came and he had to surrender. The catheterism became intolerable, and the urine, in addition to the pus, was rarely free from blood, and had a putrid odor.

In November I performed perineal urethrotomy, and introduced a full sized india-rubber tube, which was removed at the end of three weeks. I may here remark that when the tube was removed a globular phosphatic concretion, the size of a marble, had formed at the end of it, and caused considerable laceration of the passage in its removal, showing the desirability in highly phosphatic urines of removing the tube at short intervals. From the day of the operation the bladder was washed out with a hot solution of boroglyceride every four hours, night and day, and after the tube was removed the same practice was continued through a No. 12 soft india-rubber catheter passed every four hours through the perineal wound. The effect of the operation was immediate and really magical. The pain disappeared, sleep returned, and in six weeks he could retain his water for five hours. Now he can go about and visit his friends, having perfect confidence in the capacity of his bladder and the integrity of his sphincter. His urine is clear and free from albumen.

The last case I shall refer to is interesting, as it illustrates the application of the treatment in a somewhat different condition of the bladder.

In September of 1885 I saw with Mr. White, of Warrington, a young gentleman, aged 22, suffering from what at first appeared to be an acute attack of cystitis, but which subsequently was supposed to be an instance of periprostatic abscess, bursting in the bladder. For twelve months he was treated on a regular system of bladder-irrigation through a catheter, but after a few months the urethra became so tender that smaller and smaller catheters had to be substituted, until eventually he expressed his inability any longer to stand the suffering the catheters caused him. In August, 1887, I performed perineal urethrotomy, and, at the same time, took the opportunity of exploring the bladder. The supposed condition was confirmed, a large abscess cavity opening into the base of the bladder on the left hand side being discovered. From this time (upwards of two years ago) the bladder has been regularly washed out every four hours through the perineal wound by means of a Bigelow's No. 17 catheter and a twenty-ounce brass syringe. In the intervals of the washing, the patient can retain his urine without the slightest inconvenience, and he is now in the habit of driving about, and capable of attending to business. The quantity of pus is slowly but certainly diminishing, and there still remains reasonable prospect of the abscess cavity eventually closing.

I may also mention that I have also adopted the operation with success in some cases of persistent hæmaturia of obscure origin.

The advantages of the operation are, in the first place, to enable the bladder to be completely relieved of its contents whenever desirable, and by this means to derive all the benefits of physiological rest. Secondly, it provides a channel through which we can substitute a thorough for an imperfect system of washing out the bladder, the elasticity of the membranous urethra permitting the introduction of a larger catheter than the penile urethra will admit. The size of the catheter admits a greater and more forcible volume of fluid to be used for irrigation purposes, by which means we can more effectually dislodge the adherent mucus, and remove pus and any particles of calcareous matter lodged behind the prostate, or in the cavities of any sacculi, should they exist. This reduces and economizes time and lessens the risks to the patient due to prolonged exposure. At each washing, six inches of the most sensitive part of the urethra escape the irritation produced by the passage of a catheter.

By the maintenance of the permanent opening we avoid the relapse which almost invariably takes place when the opening is allowed to close. It affords a speedy means of converting a putrid into a pure urine, and finally, from the cases I have operated upon, it would appear that all the inconveniences attending enlarged prostate are permanently arrested without incurring the risks of a really serious operation, and that nothing then remains in respect of that condition to curtail the length of an average life. In all my cases there has been perfect control over the urine after the operation.

Although the title of my subject implies the inference that I restrict this plan of treatment to confirmed and otherwise hopeless cases, this by no means represents my opinion. I have determined only to recommend what I have tried and found successful, but I think it logically follows that if the operation is successful in advanced cases, we may reasonably conjecture that it would be all the more successful if adopted earlier, as the advanced cases are undoubtedly the result of neglect in the earlier stages. So far I have been deterred from operating earlier by the reluctance of patients to undergo any operation until the intensity of their sufferings has compelled them to submit.

I further venture to claim that the advantages of this method have been extended to other conditions of the bladder. For instance, in malignant growths with periodic attacks of hæmorrhage, the retention of a perineal opening generally renders the surgeon master of the situation, as he can at any moment place the patient in a condition of collapse, and by this means arrest the hæmorrhage.

From what I have stated, it may be gathered that, although what I advocate is only, perhaps, a small advance in the direction of what has been practiced before, nevertheless, small as it is, it is sufficient to secure advantages which have never been hitherto within the resource of surgery, as it affords freedom from pain and irritation, immunity from relapses, and an escape from the miseries of an incontinent bladder in cases of enlarged prostate.

In conclusion, I wish to emphasize the chief features of this treatment: First, that the operation is undertaken with the design to establish and permanently maintain a fistula, in the closest possible proximity to the bladder, but in front of the prostate. Secondly, that the fistula can be utilized at pleasure for the dual purpose of removing the contents of the bladder, and of minimizing the inconveniences attending regular, systematic, and thorough irrigation.

I am under the impression that the cases I have cited are the first on record where the bladder has been approached through the perineum with the deliberate intention of keeping the opening permanent for the purpose of relieving the pain and misery of an enlarged prostate.—*British Medical Journal*, April 13.

---

***Dyspepsia as Reflected in the Mucous Membrane of the Upper Air-passages.*** By Beverley Robinson, M. D.—It has long been a view to which I have clung with great tenacity, that affections of the upper air-passages can not be cured unless attention be directed to adjacent or remote organs, so that they may be made to exercise their functions properly. No amount of mere local treatment, whether it be medical or surgical, will produce this desired result unless the heart, lungs, liver and kidneys are all doing their share toward helping the general weal. Whilst all these organs are important to the preservation of a healthy upper air-tract, they all sink into relative littleness as compared with the immediate influence of the stomach. Let this organ perform its work well, and the nose, pharynx, and larynx are not apt to be great sufferers from catarrhal inflammation. Let the stomach be disturbed acutely or in a chronic manner by an attack of acute dyspepsia or habitual indigestion, and immediately there is evident morbid response on the part of the pituitary, pharyngeal, or laryngeal mucous membrane. I have seen children repeatedly who, whenever their stomach became upset by errors of diet or indiscretions of cake, candy, or soda-water, had febrile excitement accompanied by more or less marked pharyngeal inflammation. This pharyngeal inflammation is ordinarly one of two distinct types. Either there is a markedly red, swollen aspect, with sensations of dryness, soreness, and pain in deglutition, or there is less of these symptoms, while the tonsils on either side show a considerable number already of white exudations, situated over or near the mouths of the follicles, and which, so far as I have been able to observe, differ in no respect from similar deposits that indicate a contagious disorder with which we are all familiar. In many children, each time they are attacked with acute dyspepsia, gastric catarrh, or "biliousness," they also have within twenty-four or forty-eight hours subsequently an attack of this membranous amygdalitis. It shows itself sometimes indeed concomitantly with the evidences of disordered digestion, and again, as I have said, we may be obliged to wait a day or two longer before the throat deposits are visible. Seemingly in the latter cases there can be little doubt that the disturbance of the stomachal digestion was the original factor in the development of the throat ailment. Now, this affection of the tonsils would not come under the heading, properly speaking, of the subject of our discussion were it not accompanied almost invariably with an extension of inflammation to the upper air-tract in one or other or both directions. Frequently the nasal passages become more or less occluded from swelling and infiltration of the turbinated bodies and the mucous membrane lining the naso-pharynx. Accompanying this condition there is a nasal intonation of voice, and when the tongue is depressed to look at the throat a mass of thick white or yellowish mucus fills up the interval between the pharyngeal wall and the margin of the soft palate. Immediately an effort of hawking takes place, so that the patient is enabled to expectorate the mass and afford himself relief. At times the pharyngeal wall is for a day or more covered with a continuous coating of viscid, sticky, adherent mucus. As this mucus drops down the pharyngeal wall some of it is prone to enter the larynx and produce a slight momentary sensation of suffocation or frequent harsh cough, until it is expelled by an effort of expectoration. Whenever, as will also occur, these attacks are accompanied by considerable hoarseness, it is the generally received impression that the child has in some unexplained manner taken cold. Now, although I believe that a not dissimilar state may occasionally occur from "catching cold," yet I am of the opinion that the digestive organs, and particularly the stomach, are, as a rule, directly responsible for these effects, always admitting that a check of perspiration, or sitting in a draught, or getting a wetting, may in a sensitive child produce acute indigestion, even though there has been no evident error in diet. To strengthen my interpretation of the cases referred to, I would lay emphasis on the fact that the best medication of a similar condition is active purgation and low diet for several days. Local medication of the nose, fauces, pharynx, or larynx is of far less importance.

Having considered the effects of acute dyspepsia in children, I would direct your attention to chronic digestive disturbances. To locate precisely in small children the seat of their suffering is frequently difficult. This statement is nowhere truer than of the stomach and bowels. There are some children, frequently sons or daughters of poor parents, not rarely of well-to-do people, who have coated, flabby, indented tongues ; habitual foul breath ; yellowish conjunctivæ ; thick, sallow-looking skin of face ; small or capricious appetite ; distended abdomen ; confined or loose bowels. These same children are constantly having vomiting attacks, stomachal or abdominal pains, and are ordinarily listless, peevish, fretful, and ill disposed to play or be amused. Here, then, is a clear picture of dyspepsia, which might, of course, readily be enlarged or elaborated. How frequently in just such cases do we hear parents state that their child is unable to rest at night because it can not breathe through its nose ; that, even when it goes finally to sleep, it is restless and uncomfortable, and awakens with a start, frightened and crying out ! Such children are liable to croupy attacks, in which hoarseness and spasmodic dyspnœa are very pronounced. Now, if we examine the nose and throat, we are apt to find some of the following conditions : In the nose the turbinated bodies are swollen and pale-looking, and frequently on one or both sides almost touch the nasal septum in more or less extensive areas ; often, indeed, there is no interval between these organs. The post-nasal space habitually contains a quantity of thick mucus, which the child can not detach by its own effort, but which spontaneously dislodges itself from time to time, and which, when it reaches the lower pharynx, is usually swallowed, thus helping much to increase the dyspepsia which already exists. The pharynx, fauces, palate, tonsils, and larynx are all more or less pale-looking and relaxed. The uvula is apt to be elongated and somewhat infiltrated. The tonsils are sometimes considerably enlarged, but not in the majority of instances. Cough is a very frequent symptom. Occasionally it is a distressing, paroxysmal cough, caused by local irritation of the larynx from the presence of mucus, or by nerve irritation, or as a reflex from the elongated uvula tickling the base of the tongue or epiglottis. When we inquire into the efficient causation of such a condition, we are obliged to confess that great ignorance of or inattention to the most ordinary dietary requirements is the source from which so much trouble arises. It is interesting to observe in this connection how absolutely regardless many people are as to whether a child's bowels are moved every day regularly, as to the amount of sugar, or butter, or rich sauce a child may get at separate meals or at odd intervals during the twenty-four hours. And yet the great importance of proper care in these respects can not be a matter of reasonable doubt to any one who has closely observed the effects of the simplest remedial measures in restoring perfect equilibrium to the system. The remedial measure may not consist necessarily in doing something actively; it may merely be essential to eliminate or get rid of that which causes the outbreak of trouble.

Taking away an undue amount of sugar from the dietary of some children will enable them to pass restful nights when, previous to this exercise of care, they would have attacks of stridulous cough which would alarm a household. The free use of lime-water, in one instance I recall, broke up very rapidly spasmodic attacks of cough and hoarseness very distressing in character, accompanied by an eruption of urticaria on the face and neck. These attacks occurred in a little girl who for two years was unable to drink a glass of simple milk without causing such an attack. If she ate a small slice of bread with the milk, the attacks referred to did not occur.

I can only explain such an occurrence by supposing that the small amount of bread helped to divide the milk cure in such a way as to prevent stomachal dyspepsia.

In adults we are able to get, of course, clear descriptions in regard to digestive ailments if manifestly present, and to trace the connection more accurately between them and affections of the mucous membrane of the upper air-tract. In many cases it is difficult accurately to determine, however, which is the occasion of most distress—the dyspepsia on the one hand, or the catarrhal inflammation of the upper air-passages on the other. Not only is the statement true, so closely correlated are the two conditions, that when one is better the other improves, and if one grows worse the other immediately follows the same march, but it is also often evident that dyspepsia prceded for a long while, in a more or less pronounced manner, the advent of disordered function of the upper air-tract. This is not, however, always the case. No matter how closely we interrogate our patients at times, we can form no definite opinion as to which disease manifested itself at first. What I have said in regard to the effect of acute dyspepsia in children is only in part true, as a rule, of adults. Men and women are far less exposed to have the *follicular* form of amygdalitis accompanying the blocking up of the nose from pituitary thickening and catarrh of the naso-pharynx. Still, such cases do occasionally occur. Among dyspeptic adults there are several familiar types of reflected inflammatory or other disturbances of the mucous membrane of the upper air-passages. These depend much upon the character or underlying causes of the dyspepsia. In women, especially those who are pale, careworn victims of nervousness, palpitations, leucorrhœa, and menstrual derangements, we shall, in my experience, often discover atrophic conditions present in the nose and throat. I am inclined to believe at present, mainly from analogy, that just as the glandular apparatus seems insufficient in the stomach to furnish a healthy and adequate amount of digestive fluids, so the pale, contracted, improperly nourished pituitary membrane, which is only an accompaniment here of gland atrophy, explains in some degree the presence of those hard, scaly, adherent, bad-smelling deposits which, under these circumstances, we are apt to notice in the nasal passages, upon the septum or turbinated bodies, or lodged high up in the naso-pharynx. * * * *—*New York Medical Journal.*, April 20, 1889.

---

***The Tabetic Eye.***—Before the Société de médecine de Paris, M. TROUSSEAU lately read an interesting paper upon this subject that appears in the *Union Médicale* for March 9, 1889. The ocular symptoms of tabes, he says, are important in exact proportion to a knowledge of their special characteristics and relations to the disease that causes them. Whether they serve to trace the malady or to confirm a doubtful diagnosis, they are equally valuable, and should receive the most careful study.

Fournier has noted eye troubles in thirty-four out of two hundred and twenty-four cases. This proportion is probably smaller than actually exists, for many patients present ocular phenomena perceptible only to the physician, and of which they themselves do not complain. These insidious manifestations are especially helpful in discovering the disease during its early stages. Certain conditions belonging to the pre-ataxic state have been set down to syphilis pure and simple, owing to ignorance concerning ataxia, and to the fact that so frequently an antecedent history of syphilis is given. According to Galezowski, in nine cases out of ten, tabetic affections of vision are due to syphilis alone. Of thirty cases investigated by the author, twenty-five presented unmistakable evidences of this disease, two were doubtful, and in the three others the patients had never been inoculated.

The eye muscles, the iris, and the optic nerve are the tracts most apt to be affected, but they are not the only ones. In 1886 Pétrolacci, of Montpellier, wrote a thesis on ataxic lacrymation. Feré had under observation a patient in whom attacks of circumorbital pains of lightning-like character and unilateral lacrymation appeared and disappeared with equal suddenness, and in ataxic subjects disseminated circumorbital anæsthesia is of frequent occurrence. Berger has called attention to the diminished ocular tension during the paralytic stage.

Ophthalmic migraine, with its scotoma and hemianopsia, the author thinks may be allied to tabes, for he has observed two patients in whom frequent attacks of this sort accompanied the pretabetic period. One of the unfortunates retained this inconvenient symptom. As a general thing, not enough importance is attached to ophthalmic migraine as a precursory sign of ataxia.

The eye muscles are often paralyzed during the course of the disease, sometimes at the beginning and sometimes toward the end. This seems to have no clinical connection with optic atrophy. In other words, it is unreasonable to conclude from the presence of muscular paralysis that the patient is predisposed to optic atrophy. In six hundred cases of atrophy, Galezowski has encountered, before or during the course of profound ataxias, two hundred in which muscular paralysis existed. This simply illustrates a certain relative frequency, but can not be looked upon as bearing a very close relation. The lesions are of a different order. The prognosis is of a fatal nature in papillary atrophy, while muscular paralyses are curable. When, however, such paralyses are joined to pupillary disturbances, it is safe to affirm the probability of tabes. In the pre-ataxic stage, when the diagnosis is doubtful, the presence of these united signs is of chief importance. Ocular paralyses vary in character according to the stage of the spinal-cord affection. At the onset they are for the most part simple forms of paresis (diplopia without strabismus), developing and disappearing readily enough, sometimes instantaneously and without treatment, and returning suddenly at times. They may affect one or several muscles at the same time or in succession. These are the peculiar phenomena formerly considered distinctive of syphilis. But they are certainly rare except in the syphilitic patient who is a candidate for ataxia.

The muscles that move the ball may be attacked, and also those of the lid, causing the slight narrowing of the palpebral fissure that Berger notes in ataxic subjects. The ptosis that exists in other cases has a similar origin, as well as the paralysis of the levators of both sides seen by Déjérine. It is impossible to fathom the cause of phenomena so *bizarre* in any brief survey of the subject. They may be due to irritation simply; to nuclear scleroses; to sclerosis of the vertebral artery and its branches, as Schmeichler suggests; or to peripheral neuritis, according to Déjérine's theory; or, let us say with Brown-Sequard, considering the flitting character of these motor paralyses, they have a cause as yet indefinite.

The pupil of the tabetic eye is a source of much valuable information. There may be single or double myosis of the pin-hole variety, rendering an examination of the fundus extremely difficult; or mydriasis may exist, most frequently on one side only. Sometimes there is paralysis of accommodation without mydriasis, in which case Galezowski has noticed circumorbital anæsthesia. In some cases light does not affect the pupil, but its reaction in accommodation is normal. In others, on the contrary, the Argyll-Robertson pupil in itself is a guide to a correct diagnosis. Berger calls attention to an elliptic deformity of the pupil. The tabetic eye is also subject to an affection of the gravest moment, to wit, atrophy of the papilla. Erb notes in it twelve out of a hundred cases; Mæli, in thirteen in a hundred; and Schmeichler finds it forty times in a hundred cases. Its chief characteristics are steady progress and the well-known appearance that the opthalmoscope reveals. The grayish color of the papilla has been alleged as a pathognomonic sign of ataxia. Clinically, this fine distinction can not be admitted. It is not rare to come across ataxic papillæ that are very white. While the papilla is progressively losing its normal color—indeed, before there is any change in the tint—there is narrowing of the field of vision, which is preceded by a diminution of color perception according to the following order: green, red, blue. At the same time time there are lacunæ and scotomata in the visual field. Abadie finds a charactertistic picture of ataxic atrophy. Charcot points out the fact that sclerosis of the optic nerve may precede typical ataxic phenomena and betoken them a long time in advance. Whatever the period of its appearance, it condemns the unhappy victim to a certain and total blindness that can not be delayed or relieved.—*New York Medical Journal.* April 20, 1889.

---

***The Radical Cure of Inguinal Hernia.***—At a meeting of the New York Academy of Medicine last February, Dr. Charles McBurney read a paper in which he reported thirty-six operations for the radical cure of hernia by a method that is essentially his own; of these cases only one relapsed, and that was due to the faulty ligation of the sac, and one patient died eight days after the operation, having developed alcoholic delirium.

The operation is essentially his, because Dr. McBurney stated that subsequent to his conception of the operation he learned that Otto Riesel (*Deutsche Med. Woch., Berlin*, 1877, pp. 449, 467) had advised similar technique to that that he employed. The paper is published, with illustrations, in the *New York Medical Record* of March 23, and from it we take the following details.

Certain fundamental principles are first insisted upon: "In the formation of inguinal hernia the first predisposing and inviting cause is the slight pouch or pocket formed by the peritoneum as it is reflected over the upper part of the spermatic cord in the male, and over the round ligament in the female, at the point where this cord enters the internal abdominal ring. The loose connective tissue which accompanies the cord, and fills the inguinal canal, forms no substantial backing to the peritoneum at this point, hence a protrusion which is at first a tiny pocket readily grows under the great and constant pressure from within to a fully developed inguinal hernia."

Assuming the correctness of the foregoing, it is necessary to remove the predisposing cause—the pouching opposite the internal ring,—and support the peritoneum at this point, in order to radically cure the hernia.

To attain these ends the operation is as follows: The patient is thoroughly cleansed, the pubes and scrotum shaved, and the bowels evacuated—if possible. A free incision is made in the skin, commencing external to the internal ring, and following the line of the canal to the scrotum. The subcutaneous tissues are incised until the sac is exposed. A blade of a blunt scissors is introduced under the edge of the external ring, and the anterior wall is slit completely up—beyond the internal ring. With fingers, or handle of the scalpel, the sac is freed from all its coverings, separated from the spermatic cord, and lifted up. The wound, and the hands of the operator are now thoroughly cleansed with some antiseptic solution, the sac opened at its distal extremity, and if intestinal contents are present they must be reduced. If omentum is present, it is securely tied with catgut, and cut off, leaving a generous stump in order that there may be no slipping of the ligature. The stump is returned to the abdominal cavity, after all adhesions are broken up.

The sac is then held up vertically from the internal ring, the operator inserting a finger through the neck into the peritoneal cavity, thus guarding absolutely against the presence of intestine or omentum. An assistant then places a ligature of strong gut, or silk, about the neck of the sac at the highest point, slight traction on the sac being made by the surgeon, and his finger being withdrawn as the ligature tightens on it. The free portion of the sac is then incised, leaving a pedicle long enough to prevent slipping of the ligature. In congenital hernia it has been necessary to sew the neck carefully and smoothly.

From four to eight silk sutures are now passed through the tissues forming the upper wall of the wound, that is, the conjoined tendon, the aponeurosis of the external oblique, including the inner pillar of the external ring, and the skin. The latter is deeply invested while each stitch is tied tightly. In the lower wall, the aponeurosis of the external oblique and the outer pillar of the ring, the fascia, and the skin, are similarly sutured, the skin being inverted. Two heavy tension sutures are passed through the skin, entering and emerging about an inch from the edges of the wound, and fastened over pledgets of iodoform gauze to prevent ulceration of the skin. The wound is irrigated, dusted with iodoform, and iodoform gauze firmly packed in the canal, thus preventing œdema of the cord and consequent interference with granulation.

The scrotal or labial wound is sewed up simply, without packing. The wound is covered with antiseptic gauze, and the abdomen and thighs firmly bandaged. A piece of rubber tissue, passed over the penis and pinned to the bandages will prevent soiling by urine. In children plaster-of-paris dressing may be applied, and in girls the bandage may be shellacked.

In ten days the dressings may be changed, but if the operation has been aseptic there will be no suppuration and irrigation of the wound will not then be necessary. Exuberant granulations may be scraped with a sharp spoon or touched with nitrate of silver. The time for healing, with the formation of a dense cicatrix, is five to six weeks. When the patient sits up, no truss nor support is to be used; as the author says, if these were required he "should look upon the operation as very far from deserving the name of 'radical cure.'"

In the discussion that followed the reading of the paper several of the more prominent New York surgeons spoke favorably of the operation, having employed it as a more desirable method than any usually resorted to.

---

*The Charity Institutions of Paris—Nursing Infants with Asses' Milk.*—In recent years, in France, conscientious efforts have been made to ascertain the principal causes of the loss of population, and it has been demonstrated by numerous facts that one of these causes consists in the physical degeneration induced by deficiency of alimentation in infancy; and the most eminent physicans of Paris, and the Director of Public Assistance, have endeavored to modify and improve the system of nutrition in the public charitable institutions, providing for recently born children lactation adequate to the necessities of the temperament and constitution.

In the Hospital for Infants' Diseases, situated in Sabres Street, there exists a section for rickety boys and girls, whose miserable aspect produces an impression of pain upon the mind—unfortunate beings who have inherited the organic vices of their parents, and who suffer from anæmia's cruel tortures.

The administration of the hospital is arranged in two separated pavilions, where there is much ventilation, with large windows that look out upon a garden, and whose walls have double rows of willow cradles perfectly equipped. The newly born receive here the personal care of the establishment, beginning with being weighed in the balance the same day they make their appearance, the operation being frequently repeated almost every month in order to determine with exactness the development of the child. The little one is subjected to an especially nutritious diet of the most tonic kind, if it had been previously fed from a refractory goat liable to convey contagious germs, it having been found by experiment that the milk of this animal, although possessing nutritive principles of the most salutary kind, presents the inconvenience of communicating by absorption the effects of those nervons accidents to which the goat is subject.

The public charities of Paris, advised by the wise doctors of medicine, have substituted for the milk of goats that of the ass, and have installed an ample yard near the pavilion of the rickety and scrofulous children, which is only separated by a short covered passage-way. Nothing is more picturesque than the spectacle of the lactation of the babies in this inclosure every morning. * * * *

The nurses, dressed in dark gowns with white caps and aprons, each carrying a child on the right arm and a little seat in the left hand, present themselves in exact turn to the women who have charge of the animals, and they hold the child, applying its lips to the teats of the docile animal. The children suck with avidity the liquid nutriment, which is fresh and of agreeable taste.

The Administration of Public Assistance of Paris has calculated that one young ass is able to lactate abundantly for a space of nine or ten months, and when this period has passed they are sold and replaced by others. It is well known that the milk of asses, by its vivifying qualities and its nutritious principles, assimilates in a great degree the milk of the nurse, and these disinherited and sick children, enjoying its beneficial effects by its permanent and methodical use, are restored little by little to health and vigor.—*La Illustracion Espanola.*—*The Sanitarian.*

---

*The Nursing Question.*—A remarkable degree of public attention has been recently concentrated upon the position of women who follow the profession of nursing. The correspondence of a lengthy and for the most part one-sided and inaccurate description which appeared upon the subject of "lady nurses" in the columns of a lay contemporary has just been followed by a virulent article, describing nurses as white slaves, in the columns of an emotional evening publication. It is not, of course, essential to pause and consider whether the article in question was the production of a professional man or woman; we may, however, assume that, whoever the author may be—man or woman—there is evidence that the facts have been obtained second-hand, and are in many important particulars exaggerated and distorted. A foolish comparison is drawn between the "white slaves" who are required to be on duty in the wards attending to the patients and the medical officers whose duties enable them to enjoy themselves at lawn tennis after their visits to the wards. In this comparison we fail to see where the hardship exists. It is essential to bear in mind that women voluntarily take office as nurses, and have plenty of opportunity of testing their physical endurance for the work before they permanently engage themselves. For their services they are well paid, and have in addition board, lodging and uniform. Except for ladies of good education and birth, who in the majority of cases, merely for private family reasons, separate themselves from their homes and embark upon a nursing career, these are advantages for the most part which are far beyond the class of persons who seek to become nurses. The number of women of good education who are now applying for the vacancies of probationers at the various nursing training establishments in the metropolis is quite unprecedented; the supply is infinitely greater than the demand in this particular sphere of woman's work, despite the hardships which are supposed to environ it. The latest development in this regard is the suggested formation of a trade-union of nurses, and a gentleman has, with this object in view, even placed himself in communication with the "overworked and underpaid *employés* of nursing institutes." Such is the wording of the announcement which he has made. We shall probably hear next of a trade-union of hospital porters, established for the purpore of compelling the various hospital authorities to supply the porters with beer to be drunk on the premises during the hours of duty. —*The Medical Press.*

---

*The Etiology of Tetanus.*—Tetanus is now generally recognized as a disease due to the ravages of a specific microbe, and M. Verneuil has pointed out that the soil contaminated by the dejections of tetanic animals preserves its virulence for long periods of time. In Havana, where the disease is rife, it has been remarked that it occurs in the vast majority of cases (132 out of 162) as a consequence of wounds of the lower extremities, and generally wherever the wound is brought into contact with the soil, as in crushed feet, comminuted fractures in which the bones are driven into the ground, and where the injuries are inflicted by agricultural implements, or dressed with earth—a common practice in rural districts. A series of experiments carried out with soil from farms where outbreaks of the disease have occurred, have afforded striking proof of the possibility of infection from this source. These data are exceptionally valuable, inasmuch as they throw a good deal of light on a disease as to the nature and etiology of which we were, until quite recently, profoundly ignorant.—*The Medical Press.*

---

***A "Cremator" Cremated.***—By the New York press telegraphic reports of March 27th, 1889, we learn that the crematory erected by Chicago for the burning of the city's garbage was burned to the ground by an incendiary fire the day before; and that citizens in the neighborhood have been indignantly remonstrating against its use for some time, alleging that it created an unbearable stench.

This is understood to be the "Distillery Crematory"—the *Mann* patent—which was so enthusiastically urged by its patrons at the Milwaukee meeting of the American Public Health Association in November last. As at that time in operation in Buffalo, it was said that the entire running expenses could be defrayed by the lubricating oils extracted from, and the residual manure of, the garbage "cremated;" and that the process was entirely free from offensive odors. The one at Buffalo has since been reported a nuisance, and that at Chicago as above. It is to be hoped that *rendering* apparatus, such as this appears to be, will not detract from or prove to be an obstacle in the way of the growing interest in garbage *cremation*, and the adoption of such cremators as will effect the purpose without nuisance.—*The Sanitarian.*

---

***Medicine in Japan.***—The Japanese are progressing in the matter of medical institutions. It is announced that in the country at the present moment there are thirty-one schools of medicine, four schools of pharmacy, and two schools of veterinary surgery. The University of Tokio, moreover, contains 1,218 students. It would be interesting to know whether the Japanese, like certain European countries, admit ladies to the classes, and if there is to be found any Japanese lady practicing in her own country. Hazarding a guess, we should feel doubtful that the Japanese have, at all events as yet, in imitation of the custom in England and other nations, placed the medical profession within the reach of their wives and daughters. In Japan European customs are often adopted, and are generally most popular, but we have no means of knowing whether lady doctors have established a *raison d'etre* in the country.—*The Medical Press.*

---

***Dried Potatoes.***—In the *Voenno-Sanitarnoie Delo*, Dr. Jakov M. Shmulevitch emphatically draws attention to dried potatoes as an important food article, possessing some very valuable advantages in comparison with the vegetable in a fresh state. The advantages claimed for the article are these: (1) While fresh potatoes easily rot, blacken, and sprout, dried potatoes, when kept duly protected from moisture, remain in the best condition for a very long time; and (2), being by far lighter and less bulky than fresh potatoes, are by far more convenient for preservation and transportation, which point has a great practical importance, especially in time of war. To be fit for culinary use, the article requires a preliminary maceration in water for ten or twelve hours.—*The Sanitarian.*

---

***Overcrowding of the Medical Profession in Germany.***—According to a foreign contemporary, the overcrowding of the profession in Germany has given rise to some apprehension on the part of the authorities. Not only does the Government refuse to open fresh schools of medicine, but quite recently the Landtag refused the subsidies hitherto accorded to the professional chairs at Halle and Marburg.—*The Medical Press.*

